# The risk factors of SARS-CoV-2 antibody level differences in healthcare workers post vaccination in Siloam hospitals: A nationwide multicenter study

**DOI:** 10.1016/j.imj.2022.10.001

**Published:** 2022-10-20

**Authors:** Allen Widysanto, Ignatius Bima Prasetya, Tandry Meriyanti, Veli Sungono, Diane Lukito Setiawan, Edy Gunawan, Bayu Adiputra, Jane Olivia Lorens, Theresia Santi, Cindy Meidy Leony Pradhana, Irawan Yusuf, Catherine Gunawan

**Affiliations:** a Siloam Hospitals Lippo Village, Banten, Indonesia; b Faculty of Medicine, Pelita Harapan University, Banten, Indonesia; c Siloam Hospitals Surabaya, East Java, Indonesia; d Clinical Research Department Siloam Hospitals Group, Banten, Indonesia; e Siloam Hospitals Lippo Cikarang, West Java, Indonesia; f Mochtar Riady Institute for Nanotechnology, Banten, Indonesia

**Keywords:** Antibodies titer, COVID-19, Immune response, S-RBD IgG, Vaccine

## Abstract

**Background:**

Several vaccines have been approved against COVID-19, and 5 have been used in Indonesia. Due to the decrease in antibody levels 3 to 6 months after the second dose of CoronaVac, healthcare workers received the third booster of mRNA vaccine (mRNA-1273) to increase the antibody level. This study aimed to evaluate the risk factors of anti-S-RBD IgG levels differences in healthcare workers.

**Methods:**

This study is a retrospective cohort study of 576 healthcare workers without previous SARS-CoV-2 infection who received 2 doses of CoronaVac and the third dose of mRNA-1273 6 months after the second dose. Blood samples were obtained 2nd, 6th, 12th, and 24th weeks after the second dose of CoronaVac vaccine administration, with mRNA-1273 booster on week 20. Quantitative measurements of IgG antibodies were performed with Elecsys Anti-SARS-CoV-2 S immunoassay. We identify the baseline factors predicting post-vaccination antibody titers using univariate and multivariate linear regression analysis.

**Results:**

This study comprised 576 participants aged 32 years old, 72.05% female, and 45.84% from high-risk occupation subgroups. The median antibodies titer level on the 2nd, 6th, 12th, and 24th weeks after the second vaccine dose administration were 40.99 u/mL, 42.01 u/mL, 54.78 u/mL, and 23,225 u/mL. Antibody levels trended highest in female and younger age group (20-29 years old).

**Conclusions:**

The third dose of vaccine increased the quantitative SARS-CoV-2 spike IgG antibody titers and eliminated differences in antibodies titer by gender.

## 1 Introduction

Within 2 years of the COVID-19 pandemic, COVID-19 vaccines were approved for use by the World Health Organization (WHO). As of 15 March, 2022 and 5 COVID-19 vaccines were used in Indonesia [1]. Vaccine efficacies (VE) ranged from 50% to 95% against symptomatic COVID-19 infections. COVID-19 vaccinations minimize asymptomatic infection and transmission and prevent severe disease, hospitalization, and death [2]. As of 15 March 2022, Indonesia's immunization coverage is 73.68%, fourth in the world [3].

Studies showed that increased antibody levels after vaccination reduce the relative risk of symptomatic COVID-19 [2]. Studies assessing antibody levels in response to vaccination, however, have shown conflicting findings. Padoan et al. found that 6 months after the first BNT162b2 vaccine, antibody levels were not linked with age or gender but with previous COVID-19 infection [4]. These results contradict other studies that have reported age- and gender-dependence of antibody levels at 6 months [5], [6], [7], [8].

Reduced antibody levels have been linked to infections and transmissions, raising concerns about long-term protection to SARS-CoV-2 and prompting the possibility of a booster vaccine [4]. Antibody levels in healthcare professionals have been seen to drop between 3 and 6 months following the second vaccine dose, with the level at 6 months being comparable to those vaccinated with 1 dosage, suggesting a gradual weakening of immunological response over time [4,6,9]. Antibody levels are reported to decrease earlier in the elderly and chronic renal disease, underweight, solid malignancy patients, and those on immunosuppressive medication, whereas they can increase in females [5,9,10]. Interestingly, some studies revealed a small percentage of patients who had antibody levels 6 months after the initial dose that were labeled "late responders" rather than statistical outliers since they developed antibodies slowly after vaccination [4,9]. The booster vaccine reduces the risk of symptomatic and asymptomatic illness, prevents transmission, and reduces viral load in infected unvaccinated people [11]. An earlier study found that the increase in antibody levels was significantly higher with heterologous regimens that included mRNA-based vaccines than with the homologous booster. Furthermore, the mRNA-1273 booster was found to be the most immunogenic and to have more reactogenicity than the BNT162b2 and Ad26.COV2.S boosters [12]. Few studies have examined booster vaccination antibody levels.

This study evaluated anti-S-RBD IgG level variations in healthcare professionals without previous SARS-CoV-2 infection following 2 doses of CoronaVac and mRNA-1273 booster 6 months after the second treatment.

## 2 Materials and methods

This retrospective cohort study was approved by Mochtar Riady Nanoinstitute for Nanotechnology (MRIN) ethical committee. The study was carried out between October 2021 and February 2022 in twelve branches of Siloam Hospitals throughout 8 out of 34 provinces in Indonesia. It included 576 healthcare professionals without previous SARS-CoV-2 infection who received 2 doses of CoronaVac (30 ug in 0.5mL of aluminum hydroxide diluent solution per dose) and a booster dose of mRNA-1273 (100 ug in 0.5 mL) 6 months after the second dose. Vaccines were injected into the deltoid muscle. Participants with a previous PCR swab test and regular antigen screening every 14 days were eliminated. COVID-19-infected patients with persisting anti-S1 IgG, anti-RBD total Ig, anti-S1 IgA, and SARS-CoV-2 neutralizing titers were eliminated [13].

Antibodies were measured on the 2nd, 6th, 12th, and 24th weeks after the second dose of CoronaVac vaccine administration, with mRNA-1273 booster on the 20th week. This study evaluated anti-SARS-CoV-2 RBD antibodies using the FDA-recommended limit for seropositivity in convalescent plasma, 132 u/mL. The kit that was used in this study is Roche Elecsys Anti-SARS-CoV-2 S on Roche Cobas 6000 (Roche Diagnostics, Mannheim, Germany). In the 2nd and 6th weeks, antibody measurements were not diluted due to limited resources, yielding maximum results range of 250 u/mL. On the 12th and 24th weeks antibodies titer was diluted 400 times, yielding a maximum results range of 100.000 u/mL. On the 24th antibodies titer was measured after the third dose (booster) administration of the COVID-19 vaccine with mRNA Moderna vaccine during the 20th week. However, there were no baseline antibodies titer measured on the 20th week due to limited resources [14].

Microsoft Excel summary data. SPSS for Windows was used for statistical analysis. Kolmogorov-Smirnov tested data normality. If *p* > 0.05, the data are normal. Normal data are displayed as mean ± SD, and abnormal distribution of data show as median (minimum-maximum). Categorical data are presented as frequency (percentage). The T-test and Mann-Whitney U-Whitney tests were used to evaluate continuous variables, and the χ^2^test was used to analyze categorical variables. If the data were not normally distributed, the Mann-Whitney or Kruskal Wallis tests would be used. The statistical significance was set to *p* < 0.05. The geometric mean titer of antibodies was calculated as the geometric mean of the positive serum samples. Multivariate analyses were performed to evaluate the correlation of antibody levels at the 2nd, 6th, 12th, and 24th weeks after the second dose of CoronaVac vaccine administration, with mRNA-1273 booster on the 20th week adjusted by age, gender, and occupational subgroups.

## 3 Results

The study was carried out between October 2021 and February 2022 in twelve branches of Siloam Hospitals throughout 8 out of 34 provinces in Indonesia. This study comprised 576 health care professionals who had received 3 doses of the vaccination and who had never been infected with COVID-19. The mean age was 31.97± 8.29. Most participants (72.05%) were female and worked in high-risk occupations such doctors, nurses, midwives, and pharmacists. (Table 1).

**Table 1:** Description demographic data of participants.

No	Variable	n	%
1	Gender		
	Male	161	27.95
	Female	415	72.05
2	Age Group		
	<30	285	49.48
	30-39	185	32.12
	40-49	75	13.02
	≥50	31	5.38
3	Occupation Subgroups		
	High risk	264	45.83
	Low risk	312	54.16

**Fig. 1:**
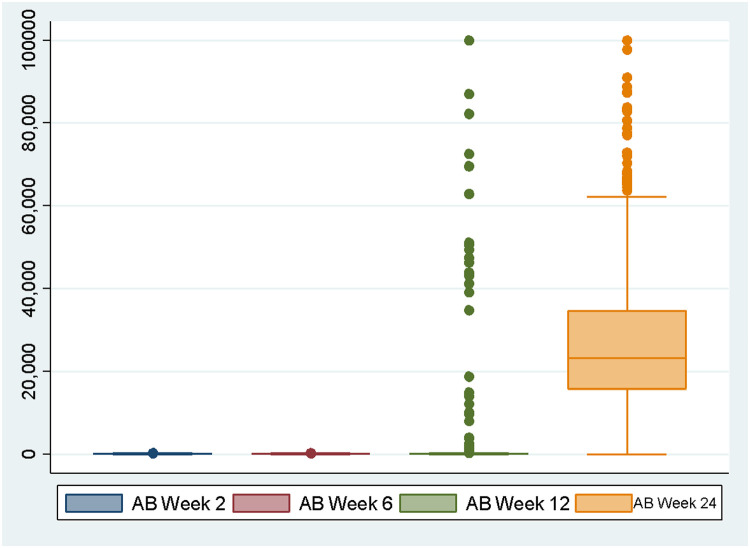
Antibodies titer on 2nd, 6th, 12th, and 24th weeks.

Antibodies were measured on the 2nd, 6th, 12th, and 24th weeks after the second dose of CoronaVac vaccine, with mRNA-1273 booster on the 20th week. The median antibodies titer level on the 2nd, 6th, 12th, and 24th weeks after the second vaccine dose administration were 40.99 u/mL (geometric mean titer (GMT) 36.09, 95% CI, 32.19–40.47), 42.01 u/mL (GMT 43.81 95% CI, 40.07-47.89), 54.78 u/mL (73.65 95% CI, 63.64–85.24), and 23,225 u/mL (GMT 19,452.05 95% CI, 17,571.05–21,534.42), respectively as shown in Table 2 and Figure 1.

**Table 2:** Serum antibody titer measured on 2nd, 6th, 12th, and 24th weeks after second dose of vaccination.

No	Variable	n	Geometric mean titer (95%CI)	Median
1	Antibody on 2nd week	576	36.09 (32.19–40.47)	40.99
2	Antibody on 6th week	576	43.81(40.07–47.89)	42.01
3	Antibody on 12th week	576	73.65 (63.64–85.24)	54.78
4	Antibody on 24th week	576	19,452.05 (17,571.05–21,534.42)	23,225

Differences between antibodies titer levels between the 2nd, 6th, 12th, and 24th weeks measurement with the McNemar test are shown in Table 3. The results were considered low level was <132 u/mL, and high-level was ≥132 u/mL. During the 6th week, 5.77% of participants who previously had low-level on the 2nd week developed an increasement to high-level. The remaining 72.22% with high level on the 2nd week (*p*-value 0.79), continued to have high level. On the 12th week, 16.35% with low level developed an increasement, and 65.72% with high level antibody on the 6th week continued to have high-level with a *p*-value <0.001. On the 24th week, 97.86% developed high level, and 100% who had high-level on the 12th week continued to have high-level with a *p*-value <0.001. (Table 3).

**Table 3:** Antibody titer levels differences among 2nd, 6th, 12th, and 24th weeks.

Antibody (u/mL)	Week 6				*p*-value	Week 12				*p* Value	Week 24				*p*-value
	<132		≥ 132			<132		≥132			<132		≥132		
	n	%	n	%		n	%	n	%		n	%	n	%	
Week 2															
<132	441	94.23	27	5.77	0.79	379	80.98	89	19.02	<0.001	10	2.14	458	97.86	<0.001
≥132	30	27.78	78	72.22		51	47.22	57	52.78		0	0	108	100	
Week 6															
<132						394	83.65	77	16.35	<0.001	10	2.12	461	97.88	<0.001
≥132						36	34.28	69	65.72		0	0	105	100	
Week 12															
<132						-		-			10	2.33	420	97.67	<0.001
≥132											0	0	146	100	

**Fig. 2:**
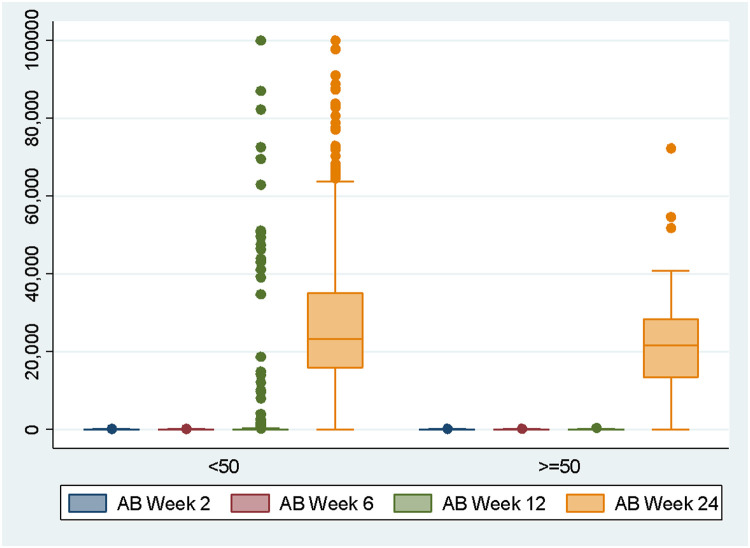
Difference antibody titer level by age.

As shown in Table 4, 5, 6 and Figure 2, median antibody titer levels were compared by age, gender, and profession subgroups. Median antibody titer levels in 2nd and 6th weeks were significantly higher in the younger age group (<50 years old) than in the older age group (≥50 years old), with a *p*-value of 0.042 and 0.044. On the 12th and 24th weeks, there were no significant median antibody titer levels differences between each group, with a *p*-value of 0.064 and 0.183. On the 2nd, 6th, and 12th weeks, median antibody titer levels were significantly higher in the female group with a *p*-value of 0.03, <0.001, <0.001. On week 24, the median antibody titer levels for male and female subgroups were 24,109 and 22,911 u/mL, respectively, with a *p*-value of 0.52 indicating no significant difference. On the 2nd, 6th, 12th, and 24th weeks, antibody titer levels in high-risk and low-risk occupational subgroups were not significantly different, with a *p*-value of 0.52, 0.46, 0.39, and 0.06.

**Table 4:** Antibody titer levels by age subgroups.

Variable	N	Mean ± SD	Min/Max	Median	*p*-value
Antibody on 2nd week					
<50 years old	545	73.31 ± 78.39	0.1/250	42.04	0.043
≥50 years old	31	61.42 ± 82.06	0.4/250	21.79	
Antibody on 6th week					
<50 years old	545	74.54 ± 76.06	0.4/250	43.08	0.044
≥50 years old	31	52.77 ± 62.24	0.4/250	29.98	
Antibody on 12th week					
<50 years old	545	1,999.78 ± 10,302.55	0.4/100,000	55.73	0.064
≥50 years old	31	75.50 ± 97.93	0.4/480.4	42	
Antibody on 24th week					
<50 years old	545	27,735.58 ± 18,970.9	5.75/100,000	23,365	0.183
≥50 years old	31	23,284.14 ± 15,269.95	0.4/72,275	21,592

**Table 5:** Antibody titer levels by gender subgroups.

Variable	N	Mean ± SD	Min/Max	Median	*p*-value
Antibody on 2nd week					
Male	161	61.65 ± 71.69	0.4/250	37.29	0.03
Female	415	76.94 ± 80.76	0.1/250	42.84	
Antibody on 6th week					
Male	161	60.69 ± 71.66	0.4/250	35.42	<0.001
Female	415	78.28 ± 76.46	0.4/250	46.53	
Antibody on 12th week					
Male	161	3,345.59 ± 13,713.79	0.4/100,000	34.08	<0.001
Female	415	1,333.93 ± 8,119	0.4/87,045	62.48	
Antibody on 24th week					
Male	161	28,535.66 ± 19,862.86	0.4/100,000	24,109	0.52
Female	415	27,092.67 ± 18,388.74	21.25/100,000	22,911

**Table 6:** Antibody titer levels by occupation subgroups.

Variable	N	Mean ± SD	Min/Max	Median	*p*-value
Antibody on 2nd week					
High Risk	264	74.68 ± 79.77	0.4/250	42.7	0.52
Low Risk	312	70.96 ± 77.62	0.1/250	40.24	
Antibody on 6th week					
High Risk	264	76.42 ± 78.24	0.4/250	44.08	0.46
Low Risk	312	70.78 ± 73.12	0.4/250	39.55	
Antibody on 12th week					
High Risk	264	1,462.21± 9,396.53	0.4/100,000	59.71	0.39
Low Risk	312	2,263.45 ± 10,538.11	0.4/82,229	51.2	
Antibody on 24th week					
High Risk	264	25,900.49 ± 16,956.6	31.63/100,000	21,801	0.06
Low Risk	312	28,846.07 ± 20,168	0.4/100,000	24,403

Multivariate linear regression of age showed significant regression with antibody titer level (log) on 2nd week. Age and gender have significant regression with antibody titer level on 6th week. Only age has showed significant regression with antibody titer level on 12th week. Among the 24th week after second dose, antibody titer level showed no significant regression with age, gender, and high-risk occupation (Table 7).

**Table 7:** Multivariate linear regression of age, gender, occupation compared with antibody titer levels.

Variable	Coefficient	*p*-value	95% CI
Antibody on 2nd week*			
Age≥50	−0.7134	0.006	−1.216, −0.21
Male gender	0.246	0.061	−0.0115, 0.504
High risk occupation	0.0327	0.782	−0.199, 0.265
Constanta	3.185	<0.001	2.734, 3.637
Antibody on 6th week*			
Age≥50	−0.5125	0.010	−0.904, −0.123
Male gender	0.3981	<0.0001	0.198, 0.597
High risk occupation	−0.0144	0.875	−0.193, 3.478
Constanta	3.129	<0.001	2.78, 3.47
Antibody on 12th week*			
Age≥50	−0.8111	0.014	−1.45, −0.1672
Male gender	0.327	0.051	−0.002, 0.657
High risk occupation	−0.102	0.499	−0.399, 0.1948
Constanta	3.8259	<0.0001	3.248, 4.403
Antibody on 24th week*			
Age≥50	−0.329	0.152	−0.7803, 0.121
Male gender	−0.023	0.844	−0.254, 0.2079
High risk occupation	0.1037	0.328	−0.104, 0.312
Constanta	9.885	<0.0001	9.48, 10.29

⁎ Transformation data of Log Antibody.

## 4 Discussions

The results showed no significant difference between antibody titer levels during week 2 and 6 measurements, which might be affected by no dilution measurement procedure. Median antibody titers were significantly different on the 2nd, 6th, and 12th weeks. There was a significant difference between median antibody titer levels on the 2nd, 6th, and 12th weeks. However, these might be affected by administering a booster vaccine on the 20th week. A study in Turkey by Uysal et al. demonstrated that postvaccine humoral immune response showed seropositivity after the 4-week postvaccine. Antibody titer levels increased significantly after the second dose of BNT162b2 mRNA vaccine in the Japanese population in the study of Takeuchi et al. [15] This research is comparable to that of Takeuchi et al., however in this study, a significantly bigger sample size was used and it was obtained from healthcare facilities located in multiple locations. This study showed antibody titer levels were significantly higher in the younger age group than the older age group on the 2nd and 6th weeks. However, there was no significant difference on the 12th and 24th weeks. Decreases in antibody titer level on the 12th week in younger and older age groups suggest the need of booster dose. These findings are similar to those produced by Shrotri et al [16], which showed waning of antibody level over 3-10 weeks after second vaccination dose, hence booster dose was suggested to be administered within 8-12 weeks after vaccination. Age was dominant factor in multivariate analysis that inversely associated with antibody titer levels. The findings are in accordance with Terpos et al. [17] in Greece reported an age-dependent pattern of immune responses less prominent in the elderly. Müller et al. [18] demonstrated that antibody titer levels were significantly lower in the elderly than in the young subject. Immune function declines with age, known as immunosenescence. In the elderly, the activity of natural killer cells and dendritic cells are reduced, and decreased variety and memory of T and B cells are seen in both the innate and the adaptive immune systems [19]. The response before the subsequent measurement could significantly predict antibody titer levels in this study.

Antibody titer levels were also observed to be considerably greater in the female group compared to the male group on the 2nd, 6th, and 12th weeks. However, there were no significant differences on the 24th week. This result is stated by recent data published by Salvagno et al. [20], who also found that females had a significantly higher response of total antibody titer level after the second dose of mRNA BNT162b2. Males with COVID-19 had a greater risk of in-hospital intubation, 3 times the odds of ICU admission, and a longer hospital stay [21,22]. Several explanations exist. Females have more CD4^+^ T cells, stronger CD8^+^ T cell cytotoxic activity, and more immunoglobulin-producing B cells than males. Females get more severe local and systemic side effects from immunizations and create larger antibody titers. Therefore, these imply that females can mount humoral immune responses more than males. Females generate more type 1 interferon (IFN), a strong anti-viral cytokine, when toll-like receptor 7 senses viral RNA than males, which is crucial for the early response to COVID-19. The X chromosome, which carries immune-related genes, is linked to increased IFN production in females [23]. Thirdly, estradiol (E2) offers an advantage against infectious disease by augmenting T cell responses and increasing antibody production, neutrophils, and monocyte/macrophage cytokine production [21,22]. Therefore, menopause is an independent risk factor for female COVID-19 patients and is associated with more severe COVID-19 due to decreased E2 [24]. In contrast, testosterone is associated with immune system suppression, age-related decline in B cells, and a trend towards accelerated immune aging in males [20,21]. These also could explain why the prevalence of autoimmune diseases is more remarkable in females with a ratio of 2:1 [23]. The lower antibodies titer level in males and older samples would suggest that this specific population may have less efficient protection against infection and an even higher risk of developing more severe COVID-19 infection. Therefore, it would be advisable for this specific population to receive a timely second vaccine dose and even a booster dose. The gender group and log antibody on 2nd and 6th weeks after second dose administration differed significantly. However, there was no significant difference on the 12th and 24th weeks. The authors suggested that the third dose of vaccine eliminates differences in antibody titer levels by gender.

Terpos et al. [17] and Levin et al. [5] reported antibody titer level decreases significantly within 4 weeks up to 6 months after the second dose of vaccine administration. However, in this study, there was a significant increase in antibody titer level measured on the 24th week, contrary to previous studies. On the 24th week antibody titer level was evaluated following mRNA-1273 booster vaccination delivery on the 20th week, therefore it is unknown if the antibody titer declined, maintained, or grew before the booster. Therefore, further studies with baseline antibodies titer measurement are recommended 6 months after the second vaccination dose and after the booster dose. No significant difference in antibody titer levels on the 2nd, 6th, 12th, and 24th weeks between the occupational group at high risk of COVID-19 transmission and low-risk groups.

The first limitation of the study is a technical problem in antibody titer that is not using dilution in the measurement process due to limited resources. Therefore, this study use the median rather than the mean to reduce the bias risk. The second limitation is the lack of baseline antibodies before the mRNA-1273 booster vaccine due to limited resources. Therefore, there was a significant increase in antibodies titer measured on the 24th week. The third limitation is that this study only uses age, gender, and occupation as the independent variable, which might lead to potential bias due to limited data to be analyzed.

## 5 Conclusion

Overall, post-COVID-19 vaccine humoral response is associated with 3 essential predictors, age, sex, and baseline serostatus. Therefore, identifying populations with a risk of inadequate post-vaccine immune response will be necessary to prevent COVID-19 infections and mortality and limit the potential of emerging SARS-CoV-2 mutation variants. Booster vaccination increased antibody levels, hence boosting with any available vaccine was better than not boosting to minimize infection and transmission. Booster protection is needed during the COVID-19 surge, as this variant seems to infect vaccinated persons. Booster immunization increases antibody titer levels, adding protection. Further investigations using baseline antibody titer measurements 6 months after the second dose of immunization and after the booster dose are recommended.

## Funding

Antibody test kit was funded by Roche Indonesia. The funder had no role in study design, data collection and analysis, decision to publish, or preparation of the manuscript.

## Author contributions

By submitting this manuscript, each of the authors indicates that he or she has had full access to all data in this study and takes complete and public responsibility for the integrity of the data and the accuracy of the data analysis.

Allen Widysanto: Concept and design, Data acquisition, Data analysis / interpretation, Drafting manuscript, Critical revision of manuscript, Statistical analysis, Supervision, Final Approval

Ignatius Bima Prasetya: Data analysis / interpretation, Drafting manuscript, Critical revision of manuscript, Statistical analysis, Supervision, Final Approval

Tandry Meriyanti: Critical revision of manuscript, Supervision

Veli Sungono: Data analysis / interpretation, Drafting manuscript, Critical revision of manuscript, Statistical analysis, Supervision, Final Approval

Diane Lukito Setiawan: Supervision

Edy Gunawan: Data acquisition, Supervision

Bayu Adiputra: Data acquisition, Supervision

Jane Olivia Lorens: Data acquisition, Data analysis / interpretation, Drafting manuscript

Theresia Santi: Supervision

Cindy Meidy Leony Pradhana: Data acquisition, Data analysis / interpretation, Drafting manuscript

Irawan Yusuf: Concept and design, Supervision

Catherine Gunawan: Data acquisition, Data analysis / interpretation, Drafting manuscript.

## Acknowledgments

We thank the laboratory and medical record staffs in Siloam Hospitals Group for their expertise and assistance throughout collecting the laboratory records.

## Declaration of competing interest

The authors declare that they have no known competing financial interests or personal relationships that could have appeared to influence the work reported in this paper.

## Data available statement

Due to ethical reasons, the raw data to reproduce the above findings cannot be shared at this time and remain confidential.

## Ethical statement

This study was approved by Mochtar Riady Institute for Nanotechnology (MRIN), Tangerang, Banten Indonesia (Protocol No. 2108017-03).

## Informed consent

Written informed consent was obtained from the patient(s) for their anonymized information to be published in this article.
