# Supplementary material for: The risk factors of SARS-CoV-2 antibody level differences in healthcare workers post vaccination in Siloam hospitals: A nationwide multicenter study
Source: Infect Med (Beijing). 2022 Oct 20;1(4):229–35. doi: 10.1016/j.imj.2022.10.001 (PMC9581641; doi:10.1016/j.imj.2022.10.001)
Supplement: Supplementary file 1 [file mmc1.docx]

**ACKNOWLEDGMENT**

Antibody test kit was funded by Roche Indonesia. The funder had no role in study design, data collection and analysis, decision to publish, or preparation of the manuscript.
