# Supplementary material for: The risk factors of SARS-CoV-2 antibody level differences in healthcare workers post vaccination in Siloam hospitals: A nationwide multicenter study
Source: Infect Med (Beijing). 2022 Oct 20;1(4):229–35. doi: 10.1016/j.imj.2022.10.001 (PMC9581641; doi:10.1016/j.imj.2022.10.001)
Supplement: Supplementary file 2 [file mmc2.docx]

**Contributor Statement**

By submitting this manuscript, each of the authors indicates that he or she has had full access to all data in this study and takes complete and public responsibility for the integrity of the data and the accuracy of the data analysis.

**Article Title:**

The Risk Factors of SARS-CoV-2 Antibody Level Differences in Healthcare Workers Post Vaccination in Siloam Hospitals: A Nationwide Multicenter Study.

We have made substantive intellectual contributions to the content of this manuscript in the following areas:

| Author Name | Concept and design | Data acquisition | Data analysis / interpretation | Drafting manuscript | Critical revision of manuscript | Statistical analysis | Supervision | Final Approval |
| --- | --- | --- | --- | --- | --- | --- | --- | --- |
| Allen Widysanto | ✓ | ✓ | ✓ | ✓ | ✓ | ✓ | ✓ | ✓ |
| Ignatius Bima Prasetya |  |  | ✓ | ✓ | ✓ | ✓ | ✓ | ✓ |
| Tandry Meriyanti |  |  |  |  | ✓ |  | ✓ |  |
| Veli Sungono |  |  | ✓ | ✓ | ✓ | ✓ | ✓ | ✓ |
| Diane Lukito Setiawan |  |  |  |  |  |  | ✓ |  |
| Edy Gunawan |  | ✓ |  |  |  |  | ✓ |  |
| Bayu Adiputra |  | ✓ |  |  |  |  | ✓ |  |
| Jane Olivia Lorens |  | ✓ | ✓ | ✓ |  |  |  |  |
| Theresia Santi |  |  |  |  |  |  | ✓ |  |
| Cindy Meidy Leony Pradhana |  | ✓ | ✓ | ✓ |  |  |  |  |
| Irawan Yusuf | ✓ |  |  |  |  |  | ✓ |  |
| Catherine Gunawan |  | ✓ | ✓ | ✓ |  |  |  |  |
