# Supplementary material for: The risk factors of SARS-CoV-2 antibody level differences in healthcare workers post vaccination in Siloam hospitals: A nationwide multicenter study
Source: Infect Med (Beijing). 2022 Oct 20;1(4):229–35. doi: 10.1016/j.imj.2022.10.001 (PMC9581641; doi:10.1016/j.imj.2022.10.001)
Supplement: Supplementary file 3 [file mmc3.docx]

**DATA AVAILABILITY STATEMENT**

Due to ethical reasons, the raw data to reproduce the above findings cannot be shared at this time and remain confidential.
